# Climate and sea surface trends in the Galapagos Islands

**DOI:** 10.1038/s41598-021-93870-w

**Published:** 2021-07-14

**Authors:** Homero A. Paltán, Fátima L. Benitez, Paulina Rosero, Daniel Escobar-Camacho, Francisco Cuesta, Carlos F. Mena

**Affiliations:** 1 Galapagos Science Center (GSC), Universidad San Francisco de Quito (USFQ), Galapagos, Ecuador; 2 School of Geography and the Environment, University of Oxford, Oxford, UK; 3 Grupo de Investigación en Biodiversidad, Medio Ambiente y Salud (BIOMAS), Universidad de Las Américas (UDLA), Quito, Ecuador; 4 Instituto BIOSFERA, Universidad San Francisco de Quito, Quito, Ecuador

**Keywords:** Environmental sciences, Climate change

## Abstract

The Galapagos Islands are a global hotspot of environmental change. However, despite their potentially major repercussions, little is known about current and expected changes in regional terrestrial climate variables and sea surface temperatures (SST). Here, by analysing existing meteorological observations and secondary datasets, we find that the Islands have warmed by about 0.6 °C since the early 1980s, while at the same time becoming drier. In fact, the onset of the wet season is currently delayed 20 days. This drying trend may reverse, however, given that future climate projections for the region suggest mean annual precipitation may increase between 20 and 70%. This would also be accompanied by more extreme wet and hot conditions. Further, we find that regional SST has increased by 1.2 °C over the last two decades. These changes will, in turn, translate into deterioration of marine ecosystems and coral, proliferation of invasive species, and damages to human water, food, and infrastructure systems. Future projections, however, may be overestimated due to the poor capacity of climatic models to capture Eastern-Pacific ENSO dynamics. Our findings emphasize the need to design resilient climate adaptation policies that will remain robust in the face of a wide range of uncertain and changing climatic futures.

The Galapagos Islands is one of the major vulnerable global hotspots to environmental and climatic change^1,2^. This is due to their unique location, which causes them to be exposed to various oceanographic and climatological variations and affects the distribution of marine species and habitats across the archipelago. In particular, the Inter-Tropical Convergence Zone (ITCZ), as well as the El Niño Southern Oscillation (ENSO), together with a complex interplay of ocean currents and winds, govern the regional climatic dynamics^3–6^. Other geophysical conditions are thought to have shaped the evolution of unique ecosystems, which are home to the emblematic species that make Galapagos famous throughout the world^7^.

Apart from influencing the Islands’ biological and ecosystem diversity, the climate—and its land–ocean impacts—also has repercussions on local socioeconomic conditions and overall human welfare^6^. For instance, oscillations in sea surface temperature (SST), due to changes in primary productivity, are often linked to fish abundance and distribution^8–12^. Such changes affect artisanal fisheries that harvest at least 68 fish species and several marine invertebrates for domestic consumption and overseas exports^13–15^.

In addition, conditions related to El Niño have been linked to a decrease in nutrient and phytoplankton concentrations and massive die-offs of terrestrial and marine species, including coral reefs^16–20^, as well as serious damages to infrastructure and major economic losses^21^. Indeed, the El Niño events experienced in 1982–83 and 1997–98 are thought to be the most intense since pre-industrial times. On the other hand, in 2016, the Islands experienced drought conditions resulting from La Niña. This delayed the onset of the rainy season, posing difficulties for agricultural activities and overall water supply. These conditions led local authorities to declare a state of emergency.

Ultimately, climatic changes will have severe implications for the Islands’ food and water security. This is of particular importance given that the Islands are also home to about 25,000 people^22^, a number that increases significantly if we also take into account the approximately 270,000 tourists who visit the islands each year^23^ (a 23-fold increase from the 11,765 tourists recorded in 1979^15^). Taken together, climate changes, along with greater stress on food, water and overall social systems, will also have repercussions for the Islands’ natural diversity and conservation strategies.

However, despite the clear role played by climate in the Galapagos Islands, little is known about the region’s present and future climatic trends^6^. This is of particular relevance for the islands of Santa Cruz, San Cristobal, and Isabela, since they are home to over 99% of the Islands’ population^24^. There is also an overall lack of understanding of current SST trends. A failure to acknowledge climate and SST changes may, in turn, severely undermine our ability to understand the extent of the fragility of human populations and ecosystems (along with their diversity) on the Islands.

The main aim of this paper is to provide an analysis of recent historical climatic observations and future projections available specifically for the Galapagos Islands. Its objectives are threefold: first, we describe the recent trends (1981–2017) for key terrestrial land surface climatological variables (precipitation and temperature) for the two Islands with sufficient hydrometeorological records (Santa Cruz and San Cristobal). To expand our understanding of key climatic variables during this period, we also include re-analysis products and satellite sources (as given by CHELSA, MODIS, ERA5, and CHIRP products; see Materials and Methods for details) to detect these trends in the region. We the estimate climatological values for Isabela. Second, we report historical SST trends for the Galapagos Marine Reserve (GMR) as read by MODIS products. Third, by examining climate projections derived from both Ecuadorian (Ecuadorian Ministry of Environment, or MAE, for its acronym in Spanish) and international climatological repositories (as given by the CHELSA projections) we shed light on the future evolution of the terrestrial-climate variables. Our analysis also seeks to provide an initial diagnostic on the dispersion of climatological datasets, highlighting the lack of sufficient observational records available.

## Results

### Current and observed temperature trends

**Figure 1:**
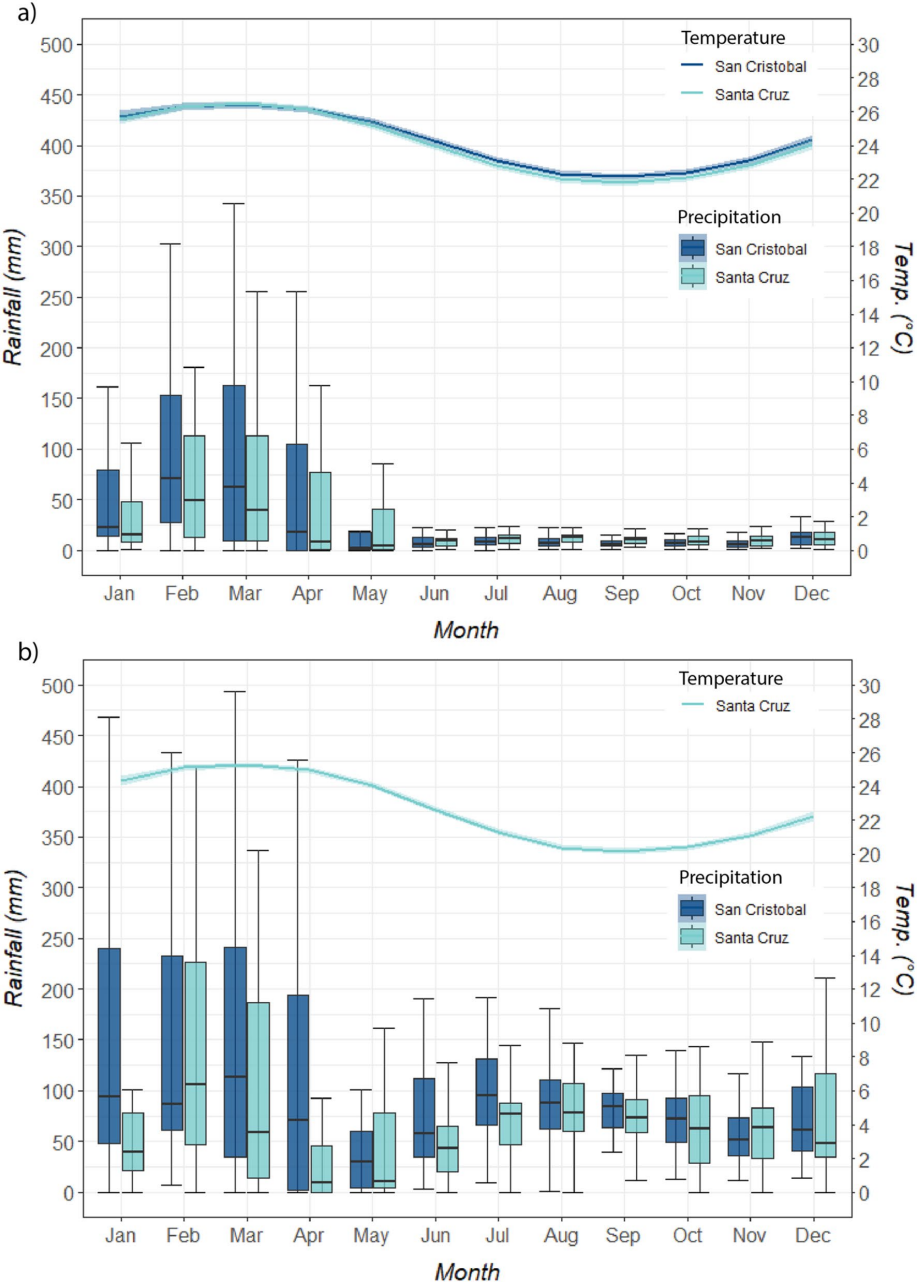
Mean annual precipitation and temperature values as observed by the meteorological stations in Santa Cruz and San Cristobal between 1981 and 2017 for: (**a**) Coastal Regions, (**b**) Highland Regions. Error bars are shown for precipitation and confidence intervals, as shaded areas, (95%) for temperature.

We find that mean annual land surface temperatures over the last 35 years (1981–2017) ranged from 22 °C to about 26 °C as read by the meteorological stations in Santa Cruz and San Cristobal (Fig. 1). Over this period, we note that mean land surface temperature has increased by approximately 0.6 °C in the lowlands (regions with altitude less than 250 m above sea level (m.a.s.l). Here the increase in mean annual temperature is approximately 0.02 °C/year, sd ± 0.4) (Supplementary Figure S1). In the highlands (regions with an altitude above 250 m.a.s.l.), mean land surface temperature has increased by approximately 0.21 °C (0.02 °C/year, sd ± 0.2). It is of particular note that, in the highlands, this increase responds mainly to higher observed temperatures during the dry/cold season (Jun–Nov). In the coastal region, or lowland areas, the pattern is reversed: here, the rate of mean temperature increases is higher during the wet/warm season (Dec–May).

We also find that the MODIS-LST satellite product seems to significantly overestimate observations (Supplementary Figure S2). ERA-5 re-analysis data seems to merely approximate observed meteorological values, and solely in the Coastal zone of Santa Cruz, while CHELSA historical temperature estimates seem to detect seasonality and magnitudes better. We also found that, on this dataset, temperature distributions show a lapse rate of 0.55 °C per 100 m. The thermal amplitude ranges from a mean air condition of 24 °C at sea level to as cold as 15 °C at 1600 m.a.s.l., at the highest, mountainous regions of the islands (Supplementary Figure S3). Also, as read by CHELSA, it can be observed that over the recent decades, mean temperatures in the coastal region of Isabela were around 22.7 (sd ± 0.3), whereas in the highlands, they were around 19.8 (sd ± 0.7).

### Current and observed precipitation trends

#### Temporal trends

As for precipitation, during the 1981–2017 period, mean annual rainfall in Santa Cruz and San Cristobal was about 500 mm (sd ± 185 in the coastal-arid regions, concentrated primarily in the wet/warm season (Fig. 1). During the dry/cold season, mean rainfall was around 130 mm (sd ± 65). On the other hand, in the highlands the mean annual rainfall ranges from about 1050 mm and 1670 mm—the difference between wet/warm and dry/cold is not as accentuated as in the coastal/low areas. During the dry/cold season, water vapour from the ocean surface rises and condenses at higher altitudes, and this condensation creates fog and heavy mists in the highlands (referred to locally as *garúa*)^4,6^. In the highlands, this *garúa* may account for nearly 35% of total rainfall in July and August on the island of Santa Cruz.

Our results also show that precipitation is highly variable, particularly during the wet season. This mainly corresponds to the influence of the El Niño years (1982–83 and 1997–98) on island climate (See Supplementary Figure S1): these years were almost three times wetter than non-El Niño years (about 3000 mm of mean annual precipitation for El Niño years vs 1100 mm for non-El Niño years).

Over the period analyzed, we notice a decreasing trend in the mean annual precipitation values across the Islands. Indeed, our analysis finds a significant decreasing rainfall trend for both San Cristobal and Santa Cruz over the last decade (Table 1). This is mainly caused by significant reductions in precipitation during the wet season, which could be as high as about 140 mm (in the highlands of Santa Cruz, or 27% of seasonal rainfall). We also find that the dry season in Santa Cruz over the last decade has shown a positive wetting trend, particularly in the highlands (about 43 mm or 9% of seasonal rainfall, or 4% of the region’s total annual rainfall). However, a slightly wetter dry season may not be sufficient to compensate for the wet season losses. In fact, when compared with the 1981–2000 period, both islands were on average 45% drier during the first two decades of this century (Supplementary Table S1).

**Table 1:** Precipitation trend analysis for San Cristobal and Santa Cruz Islands for the 1981–2018 period.

Period	Annual		Wet season		Dry season	
	Q	*P*-value	Q	*P*-value	Q	*P*-value
**Coast**						
San Cristobal						
1981–1990	4.6	0.70	14.5	0.17	− 2.1	0.37
1991–2000	− 6.9	0.85	− 2.6	0.80	6.3	0.45
2001–2010	− 1.3	1.00	− 7.5	0.72	− **5.0**	**0.00**
2011–2018	− **76.5**	**0.01**	− **70.8**	**0.01**	− **4.4**	**0.01**
Santa Cruz						
1981–1990	− **35.9**	**0.06**	− 10.6	0.34	− 2.5	0.25
1991–2000	− 26.2	0.48	− 23.8	0.43	− 1.7	0.70
2001–2010	5.2	0.25	8.4	0.27	− **3.2**	**0.00**
2011–2018	− **58.6**	**0.00**	− **61.2**	**0.00**	*5.9*	*0.00*
**Highlands**						
San Cristobal						
1981–1990	− 47.6	1.00	25.7	0.64	− 20.1	0.36
1991–2000	− 40.5	0.70	14.8	1.00	10.0	0.20
2001–2010	13.3	1.00	25.5	1.00	0.6	0.58
2011–2018	− **132.7**	**0.00**	− **39.2**	**0.07**	− **75.5**	**0.00**
Santa Cruz						
1981–1990	–	–	–	–	–	–
1991–2000	− **55.1**	**0.09**	− 34.5	0.42	− 6.4	0.64
2001–2010	− **6.2**	**0.10**	7.0	1.00	− **27.0**	**0.00**
2011–2018	− 95.5	0.30	− **138.1**	**0.03**	*43.4*	*0.04*

Trends were obtained from a Modified Mann–Kendall’s Test (MMKT) to investigate the presence of monotonic trends (upward or downward)^25^. Results of trend tests are reported at a 90% confidence interval. Marked cells correspond those where a significant trend was found. Bold mark periods where a drying trend is observed, whereas those italics where a wetting trend is detected.

It is nonetheless important to recognize the influence of strong El Niño events in these trends. When we remove the 1982–83 and 1997–98 events from the time series, various of the drying trends described above are reversed. For instance, without ENSO events, rainfall in fact increases by over 40% during the last decade in the highland of San Cristobal (Supplementary Table S1). Trends may thus be biased by the influence of unusual and very wet ENSO events during the 1982–1983 and 1997–1998 periods.

**Figure 2:**
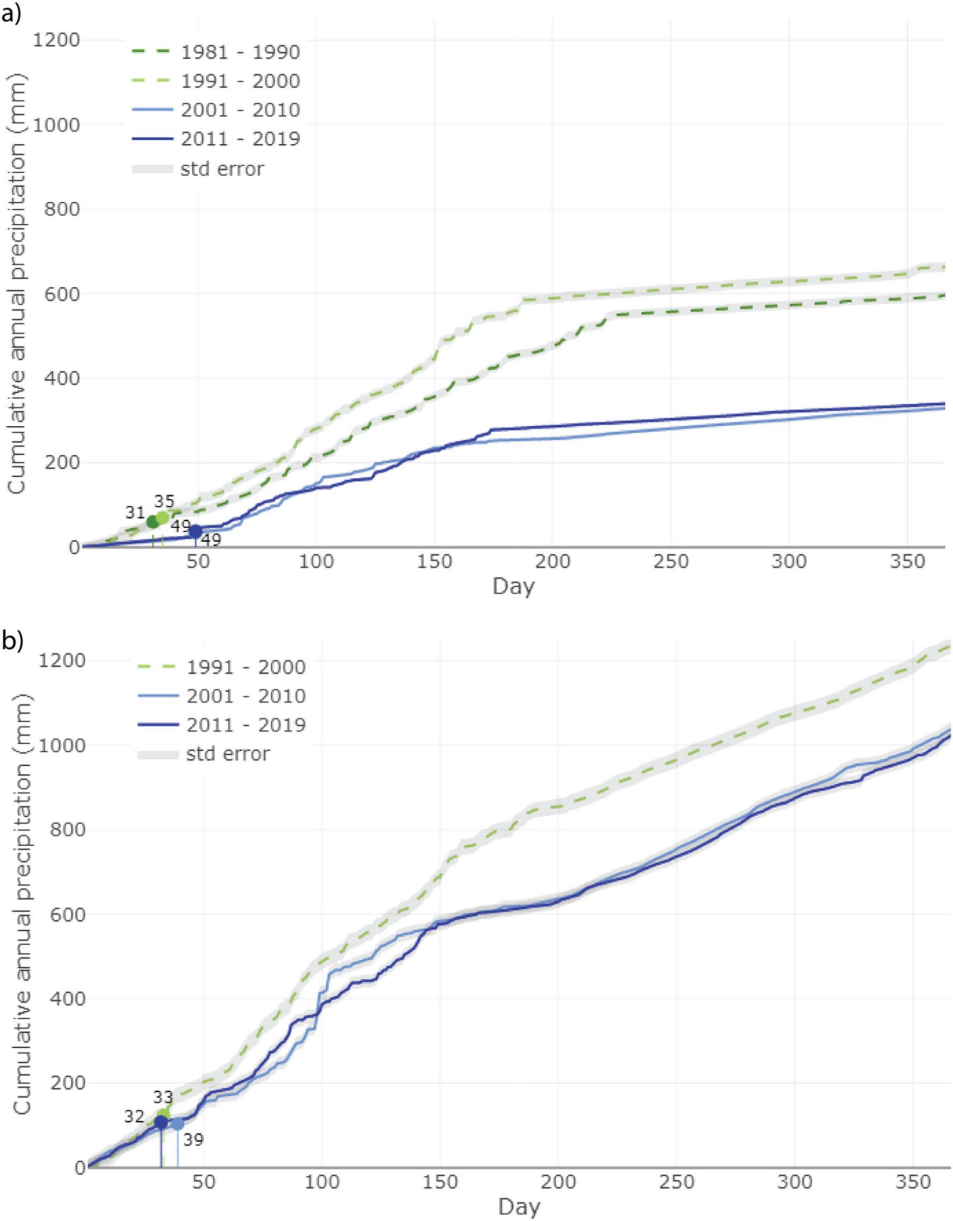
Onset of the rainy season over the (**a**) Coastal and (**b**) Highland Regions of Santa Cruz. The onset of the rainy season is defined as the day since December 1st (theoretical beginning of the rainy season) when the cumulative 10% precipitation of the total annual rainfall was achieved. Dots show the day where the 10% of the mean total annual precipitation for a given decade is achieved. Shaded areas represent standard error.

This is particularly important since the wet season seems to be typically responsible for up to 75% and 55% of annual precipitation in the coastal and highland regions, respectively, of Santa Cruz. We can also identify a decreasing trend at the beginning of the rainy season (Fig. 2) in Santa Cruz. In fact, we find that over the past two decades, a delay of almost 20 days can be observed in the rainy season onset in the coastal region. This is more acute if we also consider the extension in the number of days it presently takes to reach the 10% of accumulated precipitation, which was usual in the final decades of the twentieth century (around 80 mm). While in the 1980s and 1990s it took only around 35 days to reach this threshold, the 2000’s it has taken over 70 days. In the highlands, the delay in reaching 10% of the annual accumulated precipitation is currently about 7 days (32 days vs 39). However, the number of days it takes to achieve 10% of the annual precipitation common in the 1990s (32 days) at present takes about 45 days. However, we also find that current precipitation in the dry/cold season (from approximately day 180 onwards) and especially in the highlands—where the *garúa* maintains humidity levels—has decreased in magnitude compared to the twentieth century, confirming the drying trends described above. These results also suggest that the rainy season is both becoming drier and starting later.

#### Spatial trends

We find that the reanalysis precipitation from ERA5 product (Supplementary Figure S2) better approximates those read by the meteorological stations in Santa Cruz and San Cristobal. Nevertheless, this dataset seems to underestimate precipitation estimates in San Cristobal and appears to overestimate precipitation in the coastal areas of Santa Cruz. On the other hand, when comparing the meteorological precipitation observations with the CHELSA historical datasets and CHIRPS satellite observations, we find that these two sources do not adequately capture annual mean precipitation magnitudes and seasonality. The later seems to adequately detect seasonality, yet its magnitudes are largely underestimated. In Isabela, ERA5 estimates that total annual precipitation in the highlands typically ranges from about 565 mm to about 855 mm, while on the coasts, it ranges from about 580 mm to about 740 mm.

### Future climate

**Figure 3:**
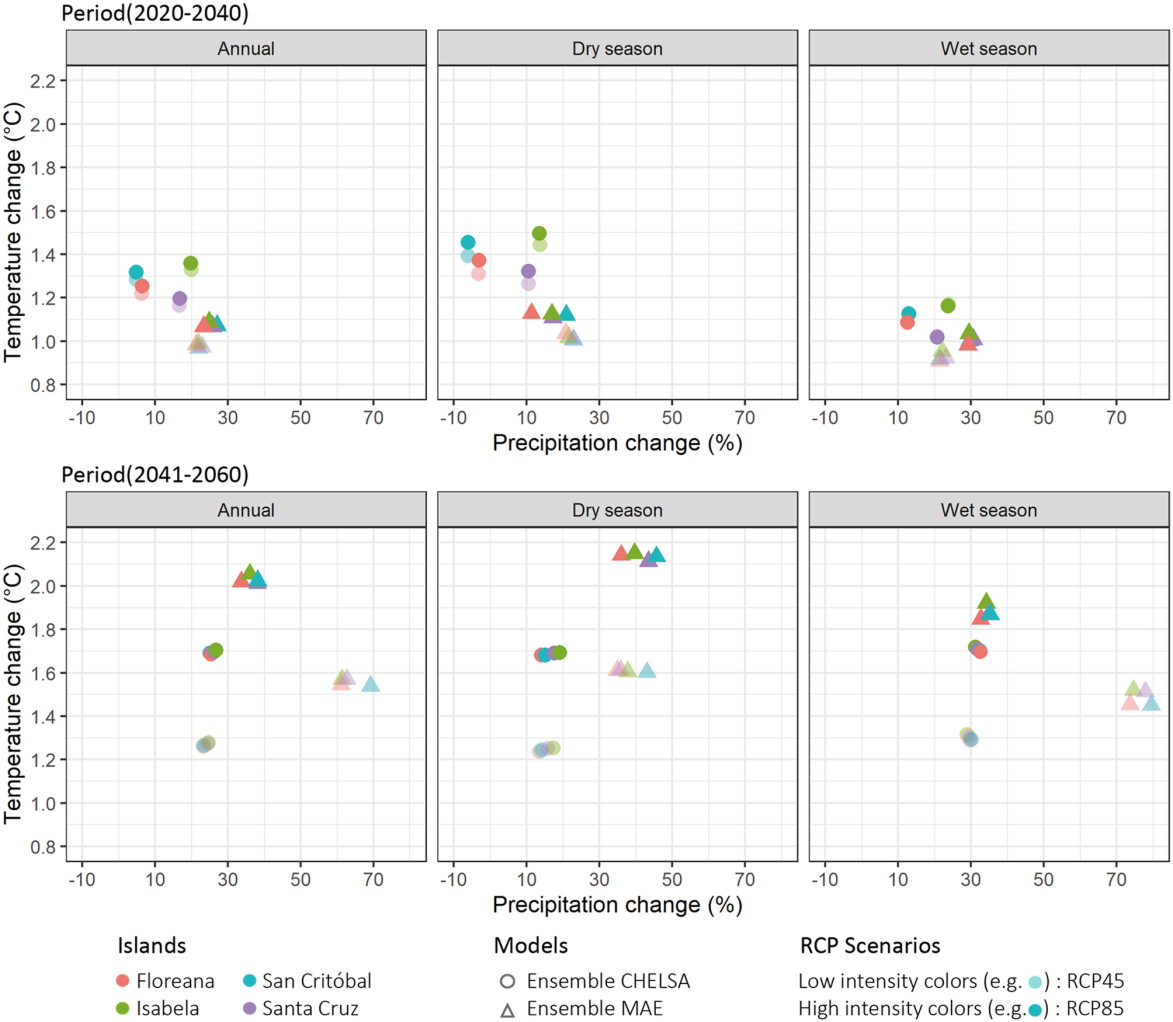
Summary of climate projections for projected average temperature and precipitation changes relative to the historic baseline as simulated by the MAE and CHELSA modelling efforts. Temperature on the y-axis represents absolute change in temperature. The x-axis represents percentual changes in precipitation. Circles represent outputs from the CHELSA modelling projections used here, and triangles represent those from the MAE experiments. The saturation of colors determines the RCP climate scenario; low color intensities represent RCP4.5, while high color intensities represent RCP8.5

In terms of future climatological patterns for the Islands, in general, the Ecuadorian, MAE, and CHELSA climate projections suggest a consistent future warming trend and wetting conditions on the three main islands studied, which is consistent across the Galapagos Islands (Fig. 3, and Supplementary Figure S5). The MAE multi-model ensembles project increases in average annual precipitation of between 30 and 45% across the Islands by 2050, suggesting a wetter future. Spatially, estimated increases in annual rainfall are accentuated on San Cristobal. As for temperature, we find that the total ensemble of climate projection estimates increases between 1.4 and 1.9 °C by the 2050-time horizon for RCP 4.5 and RCP 8.5, respectively.

However, the specific magnitude of the projected changes differs across modelling efforts, scenarios, and decades. Conservative estimates (typically RCP 4.5) project an increase in mean annual temperatures of just 0.5 °C for the next two decades (both MAE and CHELSA projections, when compared with the historical reference period) (Fig. 3). Meanwhile, the most extreme climate projections (RCP 8.5) suggest that temperatures may increase up to 2.5 °C in the period from 2040 to 2060. The MAE’s output typically simulates these extreme future conditions, while CHELSA projections estimate a maximum temperature increase of just about 1.8 °C by said time period. Likewise, as expected, the RCP 8.5 scenarios lead to greater levels of warming than CP 4.5, a difference which would be accentuated during the 2040–2060 decades.

As with temperature, precipitation projections differ across models. We find that the multi-model mean annual precipitation over the next two decades would increase between 5% and around 25% across the three islands evaluated, and indeed, throughout the overall region (Fig. 3). In general, MAE estimates for the future appear to be at the higher end of the overall ensembles, whereas CHELSA precipitation increases seem to be more conservative. This pattern seems to result from an intensification of the wet seasons, since relative increases increase in accordance with the annual estimate (in contrast to the dry season, where some scenarios even project a reduction in precipitation). For the decades between 2040 and 2060, precipitation may increase even more: climate projections suggest that rainfall in the Galapagos may increase between 20 and 70% (Fig. 3). Similarly, MAE projections generally estimate a wetter future, particularly for RCP 4.5 scenarios (as opposed to RCP 8.5 scenarios). According to these projection subsets, by the end of the century, wet season conditions may intensify by up to 80%.

**Figure 4:**
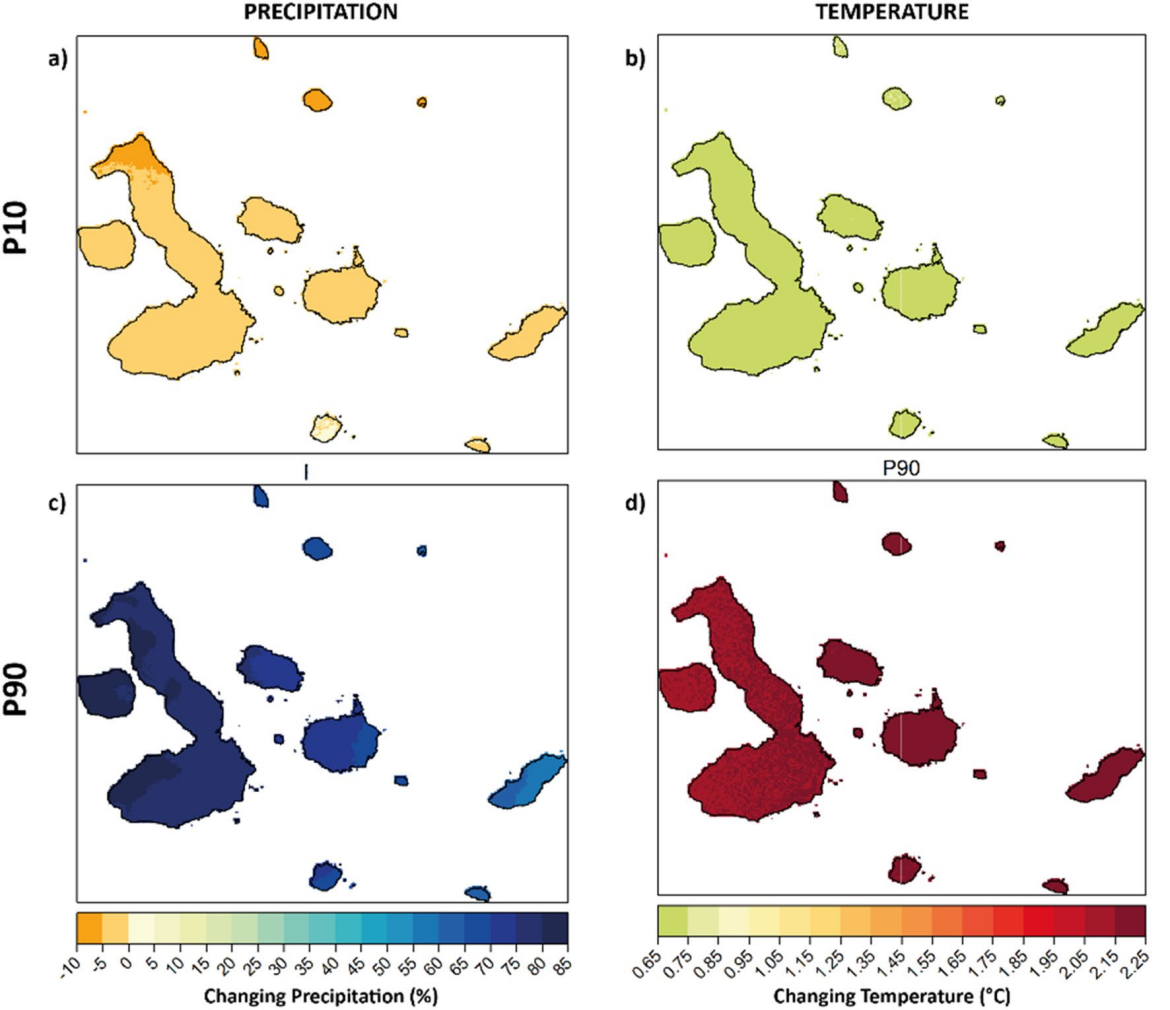
Multi-model ensemble changes in extreme wet and dry anomalies in the Galapagos Islands for the 2040–2060 period. Upper panels show multi-model changes in precipitation (**a**, %) and temperature (**b**, °C) for the 10th percentile (P10, extreme dry and cool anomalies). Lower panels show multi-model changes in precipitation (**a**, %) and temperature (**b**, °C) for the 90th percentile (P90, extreme wet and hot anomalies).

Lastly, we also calculate changes in extreme conditions in the islands (Fig. 4). Temperature anomalies indicate that for the lower percentile (10th percentile) of the ensemble of scenarios and models used here, temperature increases range from 1 and 1.5 °C (Fig. 4b). This result suggests that cooler days in the Galapagos Islands may become hotter. In the case of the upper percentile (90th percentile, Fig. 4d), the estimations show an increase in temperature between 1.5 and 2.5 °C, meaning that warm days would become hotter. Overall, these results suggest the more frequent occurrence of abnormally hot conditions, which could then translate into more common heatwaves.

Magnitudes of extreme wet anomalies (90th percentile) increase between 60 and 85% for the 2040–2060 period (Fig. 4c). This is particularly evident on Isabela, where extreme precipitation may typically increase by about 70%. As for extreme dry conditions (10th percentile, Fig. 4a), we find that the low precipitation is likely not to significantly change (percentual changes between − 5% and + 5%). However, we note that the northern parts of the three islands analysed here, and indeed the Archipelago, extreme dry anomalies would become at least 5% drier.

While we acknowledge the existence of other more sophisticated techniques and metrics to calculate extreme precipitation and temperature characteristics, our objective here is to provide an initial overview of these types of hydrometeorological conditions.

### Current and observed sea surface temperature trends

**Figure 5:**
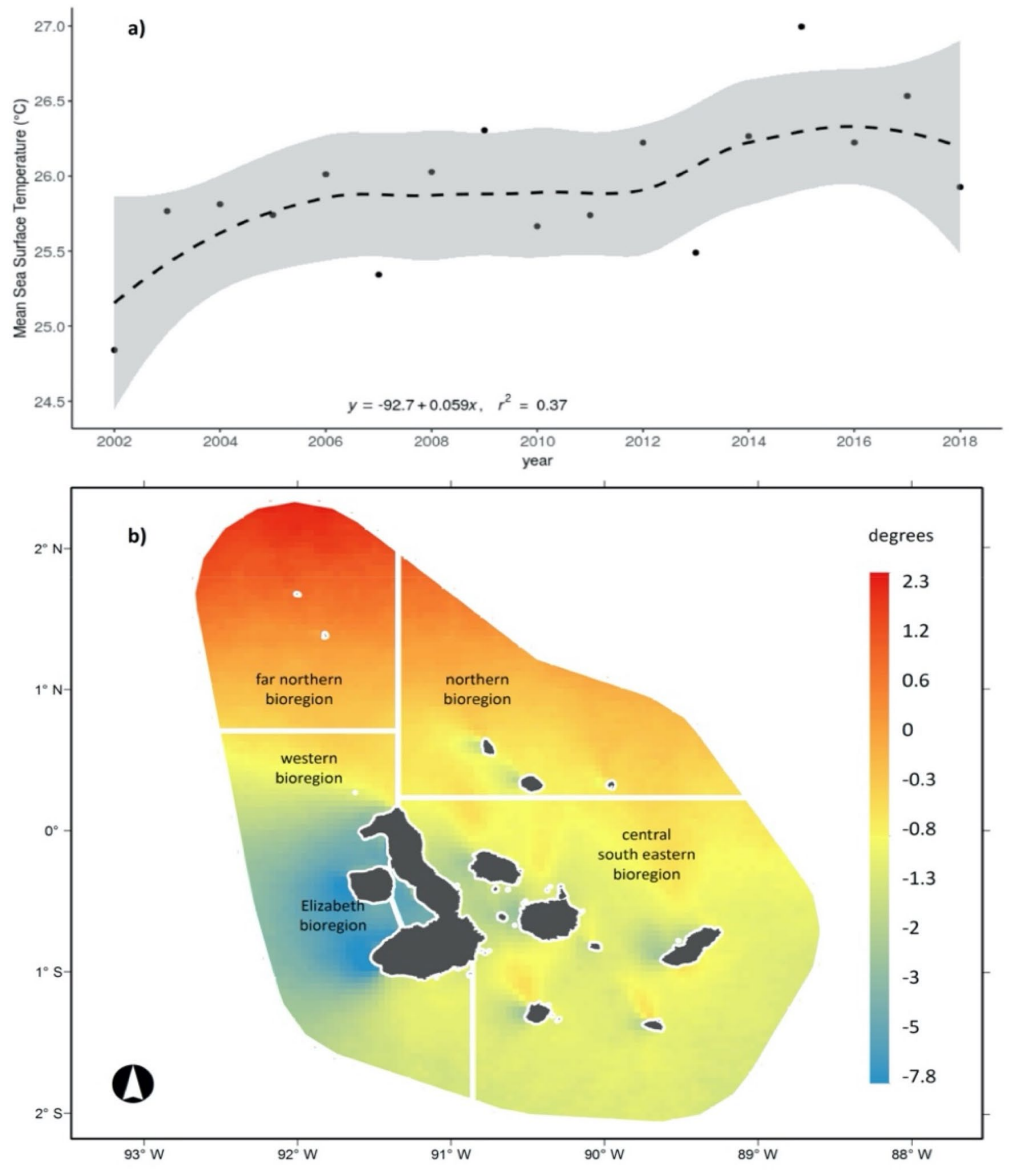
(**a**) Mean Sea Surface Temperature (SST) in the Galapagos Marine Reserve (GMR) derived from the SST MODIS L3 product for 2002–2018. Line represents the fitted linear regression between observed mean SST in the GMR for each year. The shaded area represents the 95% confidence interval (**b**) mean annual anomalies of Sea Surface Temperature (SST) in degrees for the period 2002–2018. White lines represent the five marine bioregions of the GMR as defined by Edgar et al.^26^.

In the Marine Reserve area, our results show that SST has increased at a rate of 0.06 °C per year over recent decades (Fig. 5a). For the 2002–2018 period there was an overall increase of 1.2 °C. We also observed high spatial and temporal heterogeneity in the SST throughout the Galapagos Marine Reserve (GMR). The Western, Central and Southern regions of the GMR showed cooling anomalies over the last two decades, whereas the Northern, the far Northern and some coastal areas of the Eastern and Central regions showed warming anomalies for the year 2002 (Fig. 5b).

Likewise, the interannual changes observed in satellite-derived patterns showed both the spatial and temporal variability between cool and warm zones (Supplementary Figure S6). Our analysis revealed warmer SST transitions for the periods 2002–2003, 2007–2008, 2010–2011, 2013–2014, and 2016–2017, demonstrating that the gap between cooling and warming phases has decreased over the observed period. Indeed, we find that following the warming peak registered in 2015 and La Niña of 2016, a widespread warming trend was observed in the Central-South-Eastern region for the remaining of the period studied (Supplementary Figure S6).

**Figure 6:**
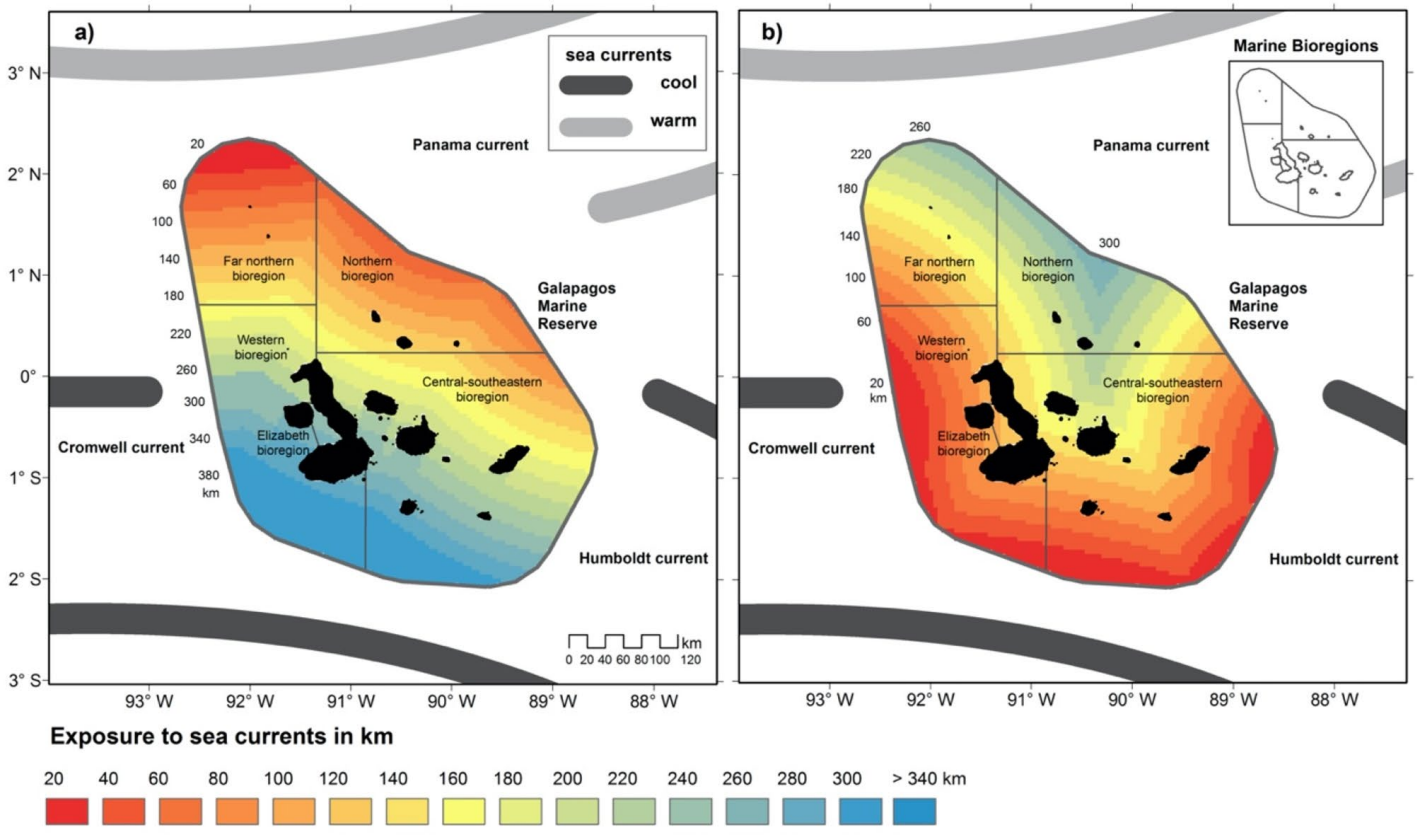
Annual exposure of the Galapagos Marine Reserve (GRM) to warm (**a**), and cool (**b**), currents. Black lines represent the five marine bioregions of the GMR as defined by Edgar et al.^26^.

Simultaneously, the Mann–Kendall test confirms different warming trends in the GMR bioregions (Supplementary Table S2). We notice that the far Northern, Northern and Central-South-Eastern bioregions exhibited significant warming trends (tau = 0.362, *P*-value = 0.048; tau = 0.362, *P*-value = 0.048; tau = 0.368, *P*-value = 0.044, respectively), whereas the Elizabeth and Western bioregions reported no significant warming trends (tau = 0.185, *P*-value = 0.32; au = 0.309, *P*-value = 0.091). These results demonstrate the influence of exposure to warm and cool currents on differentiated warming trends among the GMR (Fig. 6). Multiple regression analyses show that SST in the GMR could be explained by the time period (year), the spatial distribution of SST (longitude), and the exposure to warm currents (Supplementary Table S2).

## Conclusions and implications for the Galapagos Islands

Our results explain the recent historical and future climatic trends on the Islands of San Cristobal, Santa Cruz, and Isabela. We first find that a warming trend over recent decades is accompanied by a decrease in precipitation values across these islands (particularly during the wet season). Indeed, a registered increase of about 0.6 °C is accompanied by a reduction of around 45% of total precipitation, compared to the 1980s. This situation becomes more acute considering that we also found a delay of about 20 days in the onset of the rainy season over the last decade. Drier islands will naturally have repercussions on regional water-dependent systems, but the extent to which drier conditions may affect water supply, irrigation, and overall water and food security for the settled and floating human populations on the Islands requires further investigation. A further factor to take into consideration is that sectorial competition for water in the Islands would, in turn, impact natural systems. At present, human decisions on the Islands are thought to play the largest role in shaping the regional ecosystems and landscape dynamics^27^.

However, the drying trends found here may be biased by the fact that, during the final decades of the twentieth century (as compared to the previous four centuries) El Niño events were unusually strong in the Eastern Pacific^28^. The decrease in Eastern Pacific El Niño activity during this century may explain the detected drying trends. In fact, our results founnd that if the El Niño years are not considered in our calculations, these drying trends are attenuated, or even reversed. Unusual wet periods during the late twentieth century may result in biases when seeking to understand future climatic trends: *wetter than usual* baseline conditions may in turn lead to overestimate water availability across the Islands.

Our results also provide an understanding of the future climatic conditions that may characterize the Islands. The CHELSA and MAE models consistently agree on a wetting and warming trend across the region. In fact, precipitation is projected to increase between 20 and 70% and temperature may even increase up to 2.5 °C, in comparison with the last four decades. These increments will be accompanied by augmenting hot and wet extremes, which would ultimately translate into more severe heatwaves and floods in the region.

These extreme conditions will, in turn, have significant impacts on natural and human systems. For example, increase in rainfall conditions as a result of ENSO events can trigger a substantial growth of herbs and vines and change the community structure of arid ecosystems, making them more susceptible to colonization by invasive species^29^. The increase in the prevalence of pathogens and parasites during rainy conditions can also lead to bird populations (e.g. finches and mockingbirds) being overwhelmed, resulting in lower breeding and fledging success^30^. Likewise, wetter conditions may alter plant growth and community structure, accelerate soil erosion rates, and provide better conditions for invasive species^4^. Also, previous extremely wet conditions in the Galapagos (from past El Niño events), have led to economic losses, damages to infrastructure, damages to cropland, and impacts on human lives and ecological richness, including coral disturbances and biodiversity loss^21,31,32^. Subsequent efforts will need to account for the present and future vulnerability levels of natural and social systems (including food, water, and infrastructure) to these types of extremes.

Nonetheless, these future estimates may be constrained by the ability of General Circulation Models (GCMs) to represent climate change in the equatorial Pacific^33^. In the case of the Galapagos region, major discrepancies have been reported between GCMs and observed tropical Pacific SSTs trends. These discrepancies result from the deficiencies of CMIP5 experiments in adequately capturing the Equatorial Pacific cold tongue^34^. As a result, GCM outputs for the Galapagos region are thought to overestimate the warming and wet trend^35^. In fact, the projected warmer and wetter future contradicts the recent drying trends (described previously). More importantly, if the unusual wet decades in the late twentieth century and the overestimation of future precipitation in the Galapagos Islands are not carefully addressed, this will lead to misinterpretations regarding the Islands’ water availability and hydrological processes. Further efforts should thus investigate future changes of specific drought and flood-metrics in the Islands as well as shifts in key sub-regional hydrological processes such as future highland mists (*garúa*) conditions, while cautiously addressing these constrains.

We likewise found that over the past two decades, SST within the GMR had a mean absolute increase of 1.2 °C from 2002 to 2018. This approximation is higher than the mean warming estimates for the Equatorial Pacific over the last 40 years (0.4°–0.8°)^36^, and, critically, greater increases are expected in this region due to greenhouse warming^37^. Rising SST will in turn result in increased rainfall, which would intensify the natural and human disturbances discussed previously. Previous extreme climatic events have seriously affected productive habitats in the GMR, coral reefs, and coral and macroalgae communities, as well as oceanographic features^38,39^. Higher SST will also have variable effects on fish species: for example, positive changes in temperature are expected to yield more variable tuna and yellowfin biomass^40,41^. As a whole, climatic changes will aggravate the already significant degradation of marine ecosystems caused by anthropogenic pressures^2,42^. Despite this, the response of fisheries in the Galapagos to climate change, and its consequences for regional economic activities and food security, remain poorly understood.

We also found a heterogenous warming and cooling pattern in the GMR. This, in turn, may respond to the associated convergence of the three major current systems in the Galapagos Archipelagos (the Panama, Humboldt, and Cromwell currents). While ENSO events are thought to temporarily disrupt these currents, the extent to which global warming may also affect these processes is not clear^16,43^. Ultimately, the formation of a micro region of upwelling cold water in the Western and Elizabeth bioregions of the GMR would also lead to enhanced phytoplankton concentration, thus determining marine diversity^43^.

Our results highlight the general warming trend observed in the Islands in both surface air temperatures and SSTs. This has been accompanied by a drying trend, which may be reversed given that climate projections show a hotter and wetter future. However, considering the major reported uncertainties in GCMs, such estimates should be carefully examined. At the same time, we also acknowledge other limitations and uncertainties in understanding climatological and SSTs patterns in the Islands’. They include, among others, the lack of a comprehensive, publicly available network of instruments to capture the entire geographical diversity of the Archipelagos. As such, we also emphasize the need to incorporate uncertainty and climate risk-based approaches as the bases for planning strategies in the water, food, conservation, and other climate-connected sectors in the Galapagos Islands. These strategies must be both robust in the face of a wide range of potentially uncertain climate conditions, as shown in this study, and flexible, allowing the Islands to adapt to future climatic and non-climatic scenarios that are less than uniform.

## Materials and methods

In this study, precipitation and historical observations of temperature were first obtained from the five active weather stations publicly available on the Islands, which have information spanning three decades or more. These stations are managed by the Ecuadorian National Meteorological and Hydrological Institute (INAMHI, for its acronym in Spanish) with the collaboration of the Charles Darwin Research Station (CDRS). They are divided as follows: four principal climatological stations, and one precipitation-only station (Supplementary Figure S7).

We obtained observations from the four stations located on both San Cristobal and Santa Cruz islands, since they have recorded data for a period of 30 years or more. We should note that three stations, M0192, M0191, and M0221, provided both precipitation and air temperature readings, whereas M0508 records only rainfall. The meteorological station in the coastal region of Santa Cruz (M0191) has kept records since 1965, whereas the station in the highlands of the same island has been recording data since 1988. To maintain temporal consistency between stations and ensure an adequate comparison across time, we have used the data from the 1980s onwards.

We also acknowledge the existence of other meteorological stations owned by NGOs, individuals, and private institutions. However, they are not publicly available, or lack the temporal extension required for a robust multi-year analysis. For example, the station at Isabela (M0194) began its readings in 2002, and the recorded data is only available until 2004. As such, meteorological stations on Isabela are not functional for the purpose of this study. The Universidad San Francisco de Quito’s Galapagos Science Center likewise manages five stations on San Cristobal, but their temporal availability ranges from two to six years. The lack of sufficient observations, combined with the complex topography and habitat diversity in the islands, thus prevented us from following traditional extrapolation exercises.

We also used an altitude threshold of 250 m.a.s.l. to differentiate between coastal (low) and highland regions. This threshold is first given by the estimated altitude at which condensation (and thus drizzle and heavy mist) occurs and has been identified in previous research^3,6^. This threshold has been applied for past studies, principally in the agricultural, agrodiversity, and food security sectors on the inhabited islands^44^. We acknowledge that this threshold may reflect variation if spatial variability within specific islands is accounted for. However, we believe that this altitude sufficiently captures the climatological and other physical dynamics that serve to differentiate between high and lowlands for the multi-island comparison effort.

Next, we enriched the meteorological records from INAMHI by including available satellite observations, as well as climatological reanalysis products. As such, we first evaluated how satellite products describe temperature and precipitation patterns in the islands analyzed. To carry this out, we used the Climate Hazards Group InfraRed Precipitation with Station (CHIRPS)^45^ and MODIS^46^ satellite products for precipitation and temperature analysis, accordingly. We also then evaluated ERA-Interim or ERA5^47^ temperature and precipitation products for the region.

CHIRPS is a land-only daily rainfall dataset available since 2014. Compared with all other existing precipitation databases, the principal characteristic of this dataset is the high resolution of the available data (0.05° ≈ 5 km). As with MODIS, we use the Land Surface Temperature and Emissivity (MOD11C3) product, which provides high spatio-temporal data for the “skin” temperature at 0.05 degree of resolution. ERA5, on the other hand, is a recent product that provides gridded records of precipitation and air surface temperature at high temporal resolution and with somewhat finer spatial resolutions than other gridded data products of climate variables. This product has been developed by the Copernicus Climate Change Services and the European Centre for Medium-Range Weather Forecasts (ECMWF) with a 0.1° grid resolution. The time range of the datasets used here spans a 38-year period (1981–2017) except for MODIS data, which is available from 2000 until the present.

We also acknowledge that while the products used here are already calibrated against local observations, more local applications and studies would be benefit from additional data manipulation and correction techniques. Similarly, the differing degree resolutions of the various products used should be recognized. While the cross-product comparison occurs at the grid level, our findings may be also biased by physical and climatological processes that are not captured or are oversimplified at the sub grid level.

To understand the potential impact of climate change on the Galapagos Islands, we examined temperature and precipitation from: (1) the official estimates from the MAE, to downscale climate projections from four selected CMIP5 and; (2) climate outputs from the CHELSA effort. The MAE climate projections consist of the dynamically downscaled outputs of four GCMs: CSIRO-Mk3-6-0, GISS-E2-R, IPSL-CM5A-MR, MIROC-ESM. The final spatial resolution of this product is 10 km. For more information see^48^.

CHELSA is a high resolution (30 arc sec, ~ 1 km) dynamical global climate dataset for land surface areas, which is hosted by the Swiss Federal Institute for Forest, Snow and Landscape Research (WSL)^49^. Here we use a collection of 13GCMs that have been reported to better represent and capture the Eastern-Pacific ENSO dynamics^50,51^, and thus are relevant for the Galapagos Islands. The GCMs used here are: CNRM-CM5, CSIRO-Mk3-6-0, FGOALS-g2, GFDL-ESM2G, GFDL-ESM2M, GISS-E2-H, GISS-E2-R, HadGEM2-CC, HadGEM2-ES, IPSL-CM5A-LR, IPSL-CM5A-MR, MIROC-ESM, and NorESM1-M. Climate conditions were characterized for the reference or baseline periods 1979–2013 and 1981–2015 for CHELSA and MAE data, respectively. Overall, the CHELSA and the MAE efforts provided us with a total of 55 climatic scenarios.

Changes in extreme precipitation and temperature conditions was calculated from the ensemble of models used here. This is from the total 55 scenarios combining climate trajectories and modelling efforts. Extreme anomalies (very hot, very warm, very cool, and very dry conditions) are obtained from the 90th and 10th percentile estimates for the total of ensembles. For each ensemble, change was calculated from the difference between the future projections and their historical reference periods (979–2013 and 1981–2015 for CHELSA and MAE data, respectively.)

Lastly, to assess SST variation, we used the NASA annual SST MODIS L3 satellite product for 2002–2018 with a spatial resolution of 4 km. For this, we ran a simple linear regression model between the annual mean SST of the entire Galapagos Marine Reserve (GMR) during the observed period, which provided an estimation of the increasing trend of inter-annual SST variability. Then, we estimated SST anomalies using a reference mean surface temperature of 25 °C, calculated for the GMR for the year 2002. The differences between the reference temperature and the grid values for each year were calculated. Finally, we estimated the mean anomaly for the 2002–2018 period. For this, the spatial and temporal patterns observed in the SST anomalies and trends, respectively, were analysed by a Mann–Kenndal following the Pohlert et al. approach^52^.

The test for SST was conducted independently for the five Marine Bioregions^26^ using the mean SST of the GMR per year. Because there were differentiated spatial patterns and increasing trends for each marine bioregion, we built two models of exposure to sea currents for the GMR: first, we modelled exposure to warm and cool currents with intervals of 20 km; second, we assessed the combined effect of multiple environmental variables in explaining SST trends for the observed time period through a multiple regression analysis using standard least-squares for fitting a model with annual SST values as the dependent variable. This analysis was implemented with all the SST data for the GMR. As predictive variables, time period, the exposure to warm currents, and longitude were used. Before the regression analysis was carried out, we conducted a Pearson correlation analysis between all the variables to exclude strongly correlated (Pearson correlation, r < 0.6) explanatory variables (Supplementary Figure S8). We selected the best model by minimizing the residual mean square, the Akaike information criterion (AIC), and maximizing r2.

## Supplementary Information

**Supplementary Information**.
