# Supplementary material for: Climate and sea surface trends in the Galapagos Islands
Source: Sci Rep. 2021 Jul 14;11:14465. doi: 10.1038/s41598-021-93870-w (PMC8280102; doi:10.1038/s41598-021-93870-w)
Supplement: Supplementary file 1 — Supplementary Information. [file 41598_2021_93870_MOESM1_ESM.pdf]

Climate and Sea Surface Trends in the Galapagos Islands

Supplementary Material

Homero A. Paltán[1,2*], Fátima L. Benitez[2], Paulina Rosero[3], Daniel Escobar-Camacho[3], Francisco Cuesta[3], Carlos F. Mena[2]

1 Galapagos Science Center, Universidad San Francisco de Quito (USFQ), Galapagos, Ecuador
2 School of Geography and the Environment, University of Oxford, Oxford, UK
3Grupo de Investigación en Biodiversidad, Medio Ambiente y Salud -BIOMAS -, Universidad de las Américas (UDLA), Quito, Ecuador

* Corresponding Author, email: homero.paltanlopez@ouce.ox.ac.uk

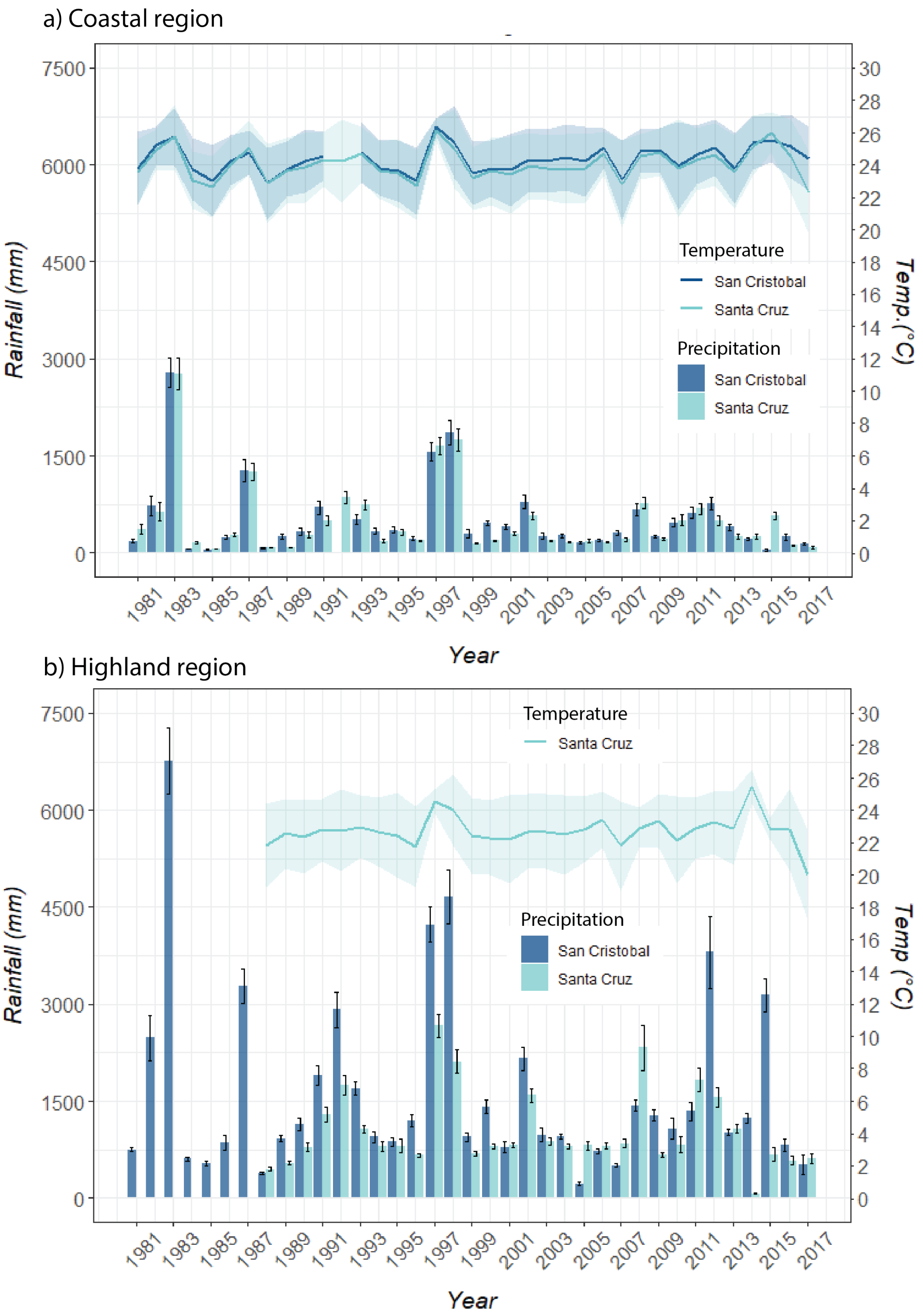

Supplementary Figure 1. Multi-annual mean precipitation and temperature values. Bars in precipitation indicate variations whereas blurred regions in temperature indicate standard deviation. Time series shown for San Cristobal and Santa Cruz as detected by meteorological stations (1981-2017).

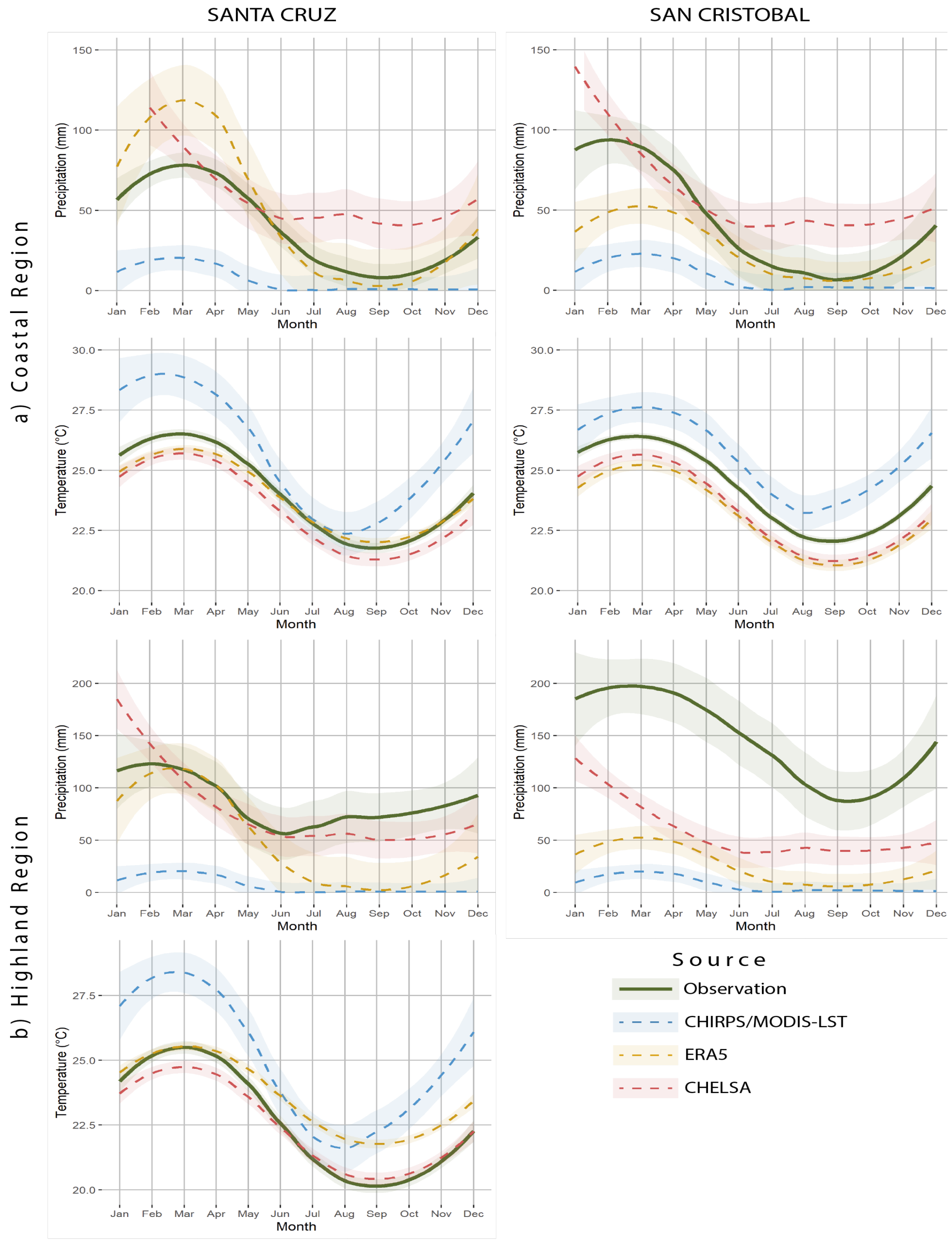

Supplementary Figure 2. Mean annual precipitation and temperature values as observed by the meteorological stations in Sant Cruz and San Cristobal between 1981-2017 and compared with CHELSA, ERA, and MODIS datasets. Shaded areas represent montly variation. Note that we do not show temperature estimates for highlands in San Cristobal since the station in this region is just pluviometric. Shaded areas indicate the confidence Interval (95%)

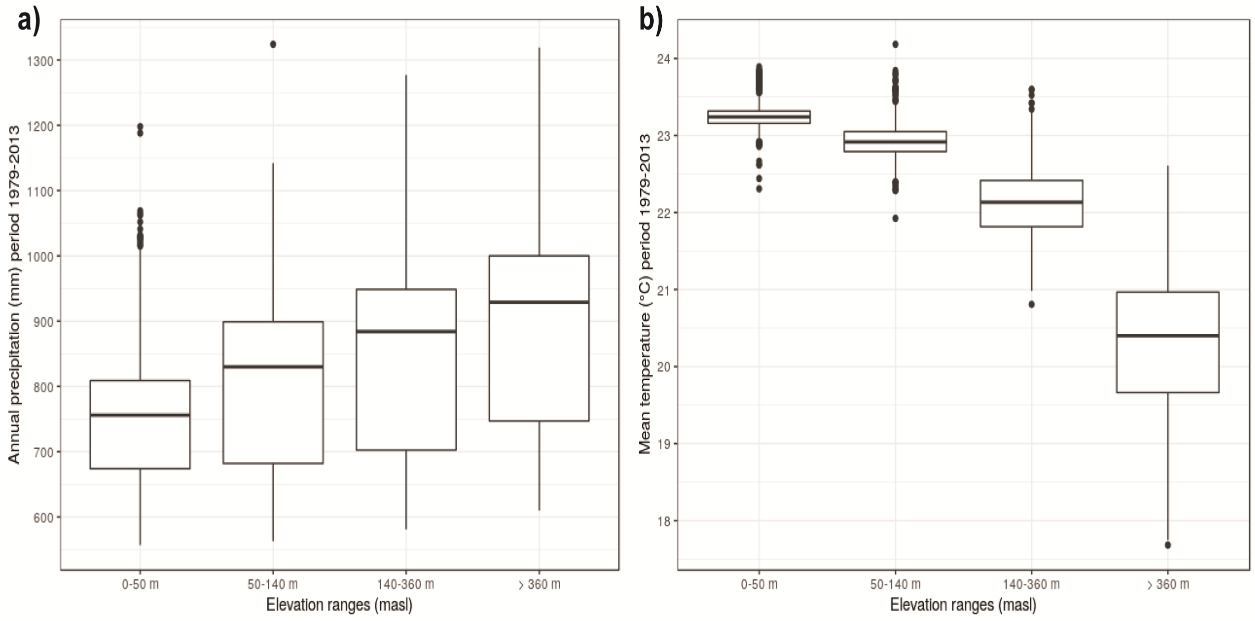

Supplementary Figure 3. (a) Annual precipitation and (b) mean air temperature for the period 1977-2013 along the elevation gradient in the Galápagos Islands. Box plots represents the interquartile range of the dataset, whereas the whiskers represent the percentiles 5 and 95 respectively. The median is shown with a line inside each box plot. Elevation ranges were defined based on quartiles frequencies derived from a 1km2 SRTM elevation model. Climate data were derived from CHELSA datasets.

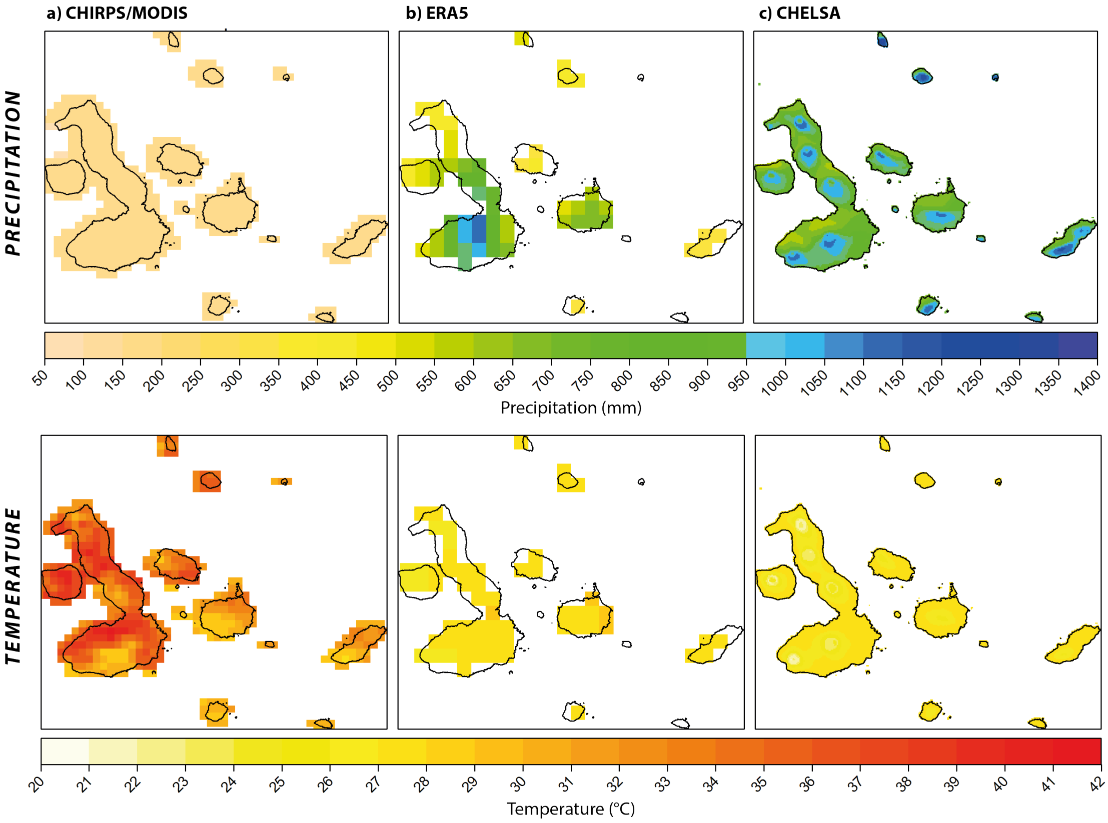

Supplementary Figure 4. Spatial distribution of annual average precipitation and temperature (mm and °C, respectively) over the Galapagos archipelago from a) satellite observations; b) ERA5 reanalysis dataset and; c) CHELSA downscaling dataset.

Supplementary Table 1. Precipitation changes (mm) in the last four decades in the San Cristobal Santa Cruz Islands. Ref. A = reference period 1981-2000; Ref. B = reference period 1981-2000 but without ENSO events.

		COASTAL			HIGHLAND		
		Prec.	Change (%)		Prec. (mm)	Change (%)	
	**Period**	**(mm)**	**Ref. A**	**Ref. B**		**Ref. A**	**Ref. B**
**SAN CRISTOBAL**	1981 - 1990	597.3	-	67.3	1770.7	-	39.2
	1991 - 2000	701.4	6.8	96.4	2077.6	6.5	63.3
	2001 - 2010	377.5	-42.5	5.7	1011.5	-48.2	-20.5
	2011 - 2017	346.3	-47.3	-3.0	1867.6	-4.3	46.8
**SANTA CRUZ**	1981 - 1990	596.8	-	67.8	no data	-	-
	1991 - 2000	653.7	0.8	83.8	1263.6	11.4	44.3
	2001 - 2010	327.3	-49.5	-8.0	1037.3	-8.6	18.5
	2011 - 2017	367.3	-43.3	3.2	985.5	-13.1	12.6

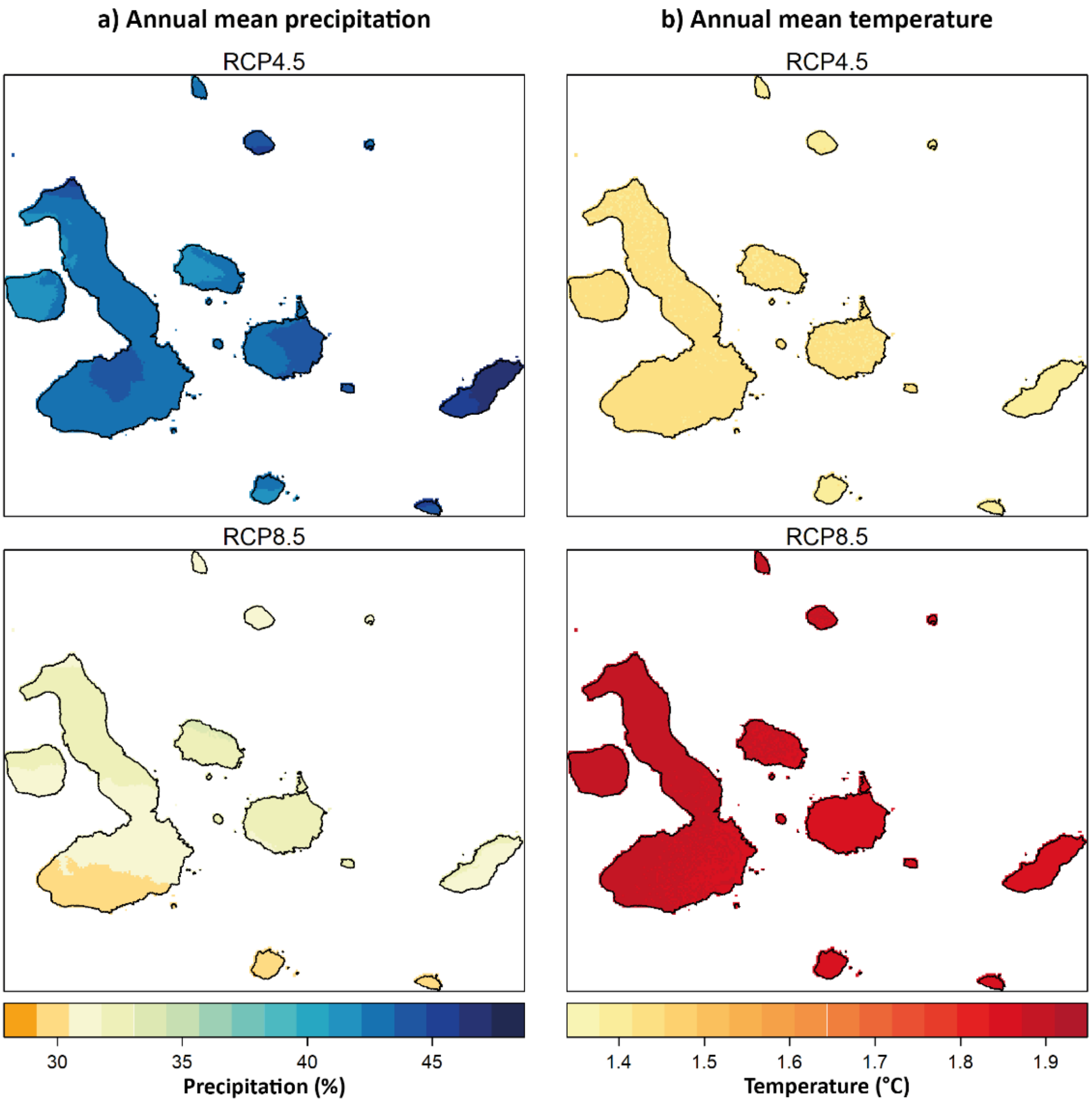

Supplementary Figure 5. Multi-model means of annual projected changes in a) precipitation and b) temperature across the Galapagos Islands by 2050-time horizon.

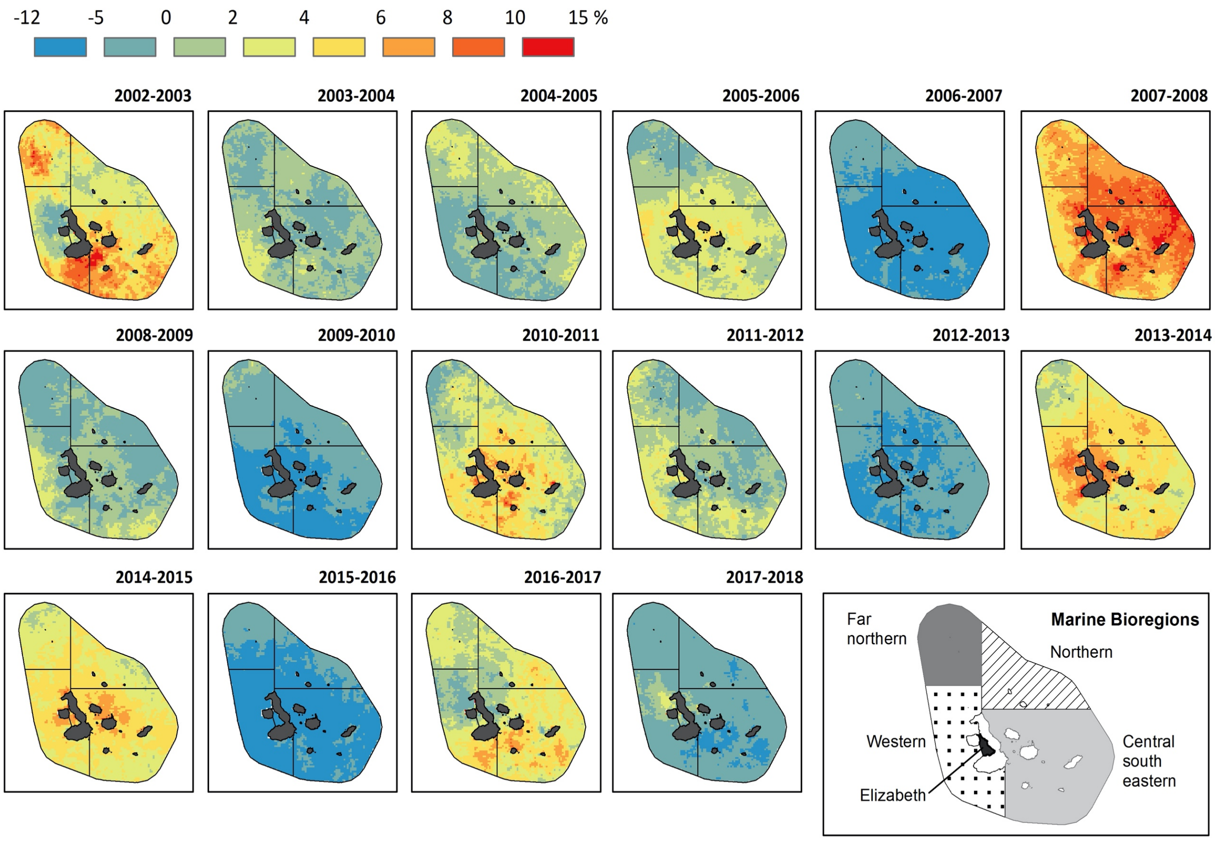

Figure 6. Inter-annual percentage of change of diurnal SST from 2002 to 2018. The plotted values are represented in discrete intervals from -5% to 10% of change in respect to the previous year. Extremes below -5% are represented in light blue, extremes over 10% are represented in red and specified below the plot of each time period respectively. The values in percentage displayed in the coloured intervals, correspond to the distribution of the observations for each time period.

Supplementary Table 2. Multiple regression model for SST for the period 2002-2018

	**Estimate**	**Std.error**	**t-value**	**Prob (>|t|)**
Intercept	25.29	0.0080	3189.580	< 2e-16
Year	0.073	0.0005	144.810	< 2e-16
Longitude	-0.308	0.0090	-32.260	< 2e-16
Dwarm	-2.732	0.0100	-272.290	< 2e-16
		**Summary of fit**		
	**r^2**	***P***	**AIC**	**RSE**
	0.470	< 2e-16	267273.9	0.8

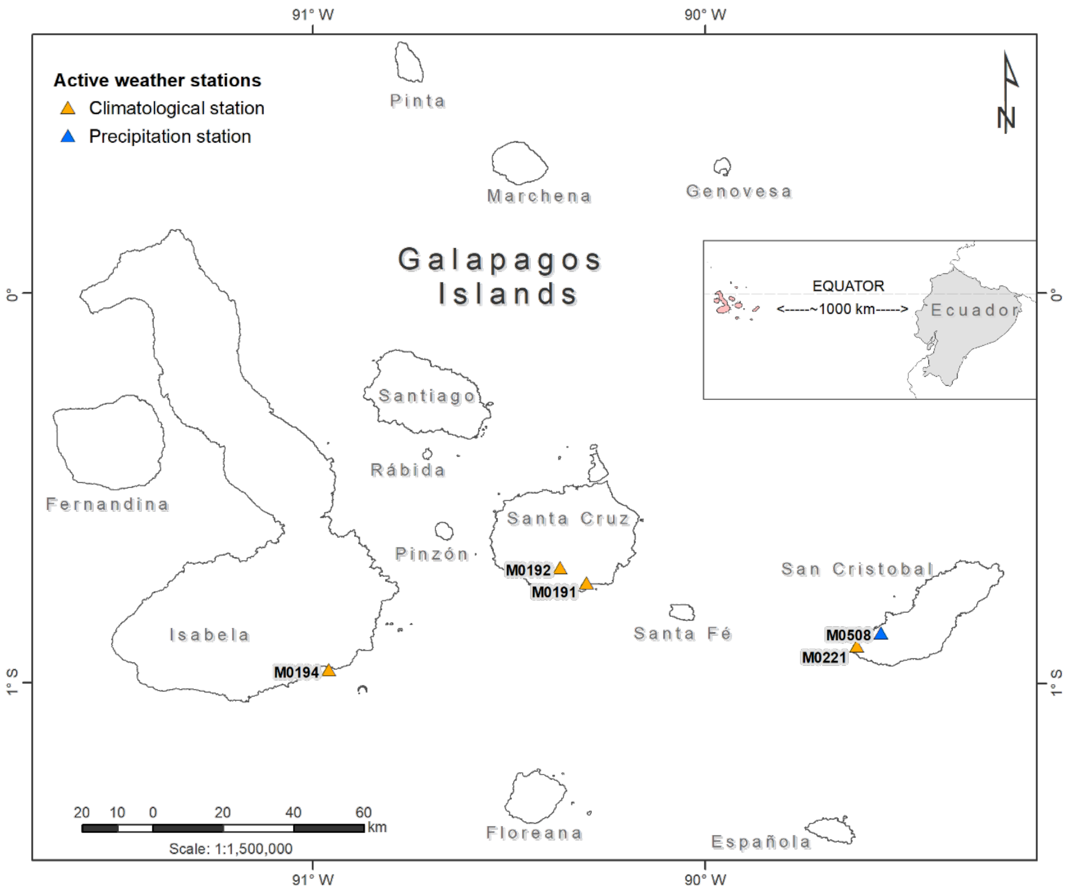

Supplementary Figure 7. Geographical location of Galapagos in relation to mainland Ecuador. Location of the active hydrometeorological stations on the islands.

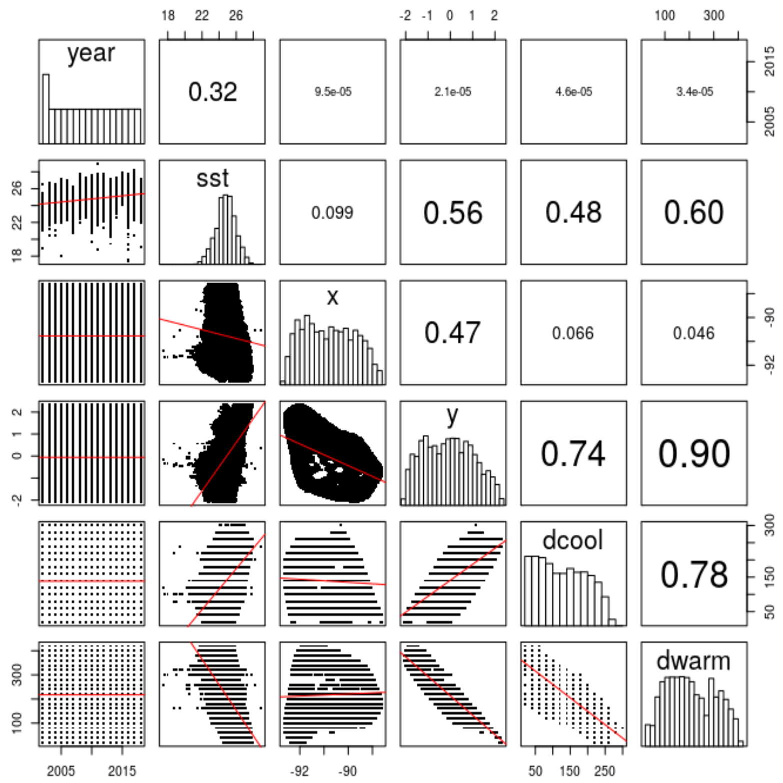

Supplementary Figure 8. Pearson correlation between SST (degrees) and explanatory variables. Latitude and longitude are expressed in decimal degrees, and exposure to warm (dwarm) and cool currents (dcool) in kilometres.
